# Effects of a Positive Psychology Intervention on Mental Health and Well-Being Among Mothers in Riyadh, Saudi Arabia

**DOI:** 10.3390/healthcare13151925

**Published:** 2025-08-06

**Authors:** Munira Abdullah AlHugail, Deemah Ateeq AlAteeq

**Affiliations:** 1 Faculty of Art, Princess Nourah bint Abdulrahman University, Riyadh 11671, Saudi Arabia; 2 Internal Medicine Department, College of Medicine, Princess Nourah bint Abdulrahman University, P.O. Box 84428, Riyadh 11671, Saudi Arabia

**Keywords:** positive psychology intervention, working mothers, gratitude, well-being, depression, anxiety, stress, Saudi Arabia, mental health, cultural adaptation

## Abstract

**Background:** Concerns over women’s mental health have intensified globally, especially among mothers managing dual careers and family responsibilities. Positive Psychology Interventions (PPIs), such as gratitude journaling and well-being workshops, have demonstrated promise in enhancing mental health; however, their applicability in Arab contexts remains underexplored. This study aims to investigate the effectiveness of PPIs on mothers’ well-being, gratitude, depression, anxiety, and stress in Saudi Arabia. **Methods:** This quasi-experimental, one-group pretest–posttest study assessed the effects of a four-week PPI on 37 Saudi working mothers (aged 21–50 years) employed at a private school in Riyadh. The intervention included guided gratitude journaling thrice weekly and two workshops on positive psychology and gratitude. Pre- and post-intervention assessments used validated Arabic versions of the Depression Anxiety Stress Scale (DASS-21), WHO-5 Well-being Index, and Gratitude Questionnaire (GQ-6). **Results:** Significant improvements were found post-intervention: depression, anxiety, and stress scores decreased (*p* < 0.001), while well-being and gratitude increased (*p* = 0.001). However, participants with lower household income (<50,000 SAR) showed less improvement, indicating a potential moderating effect of socioeconomic status. **Conclusions:** The intervention demonstrated promising short-term improvements in mental health and well-being among Saudi mothers. The findings underscore the importance of culturally appropriate PPIs and highlight the need for further research using controlled, long-term designs. Limitations include the small, non-random sample, absence of a control group, and restriction to a single geographic region.

## 1. Introduction

Mental health is a dynamic and multifaceted aspect of human well-being that enables individuals to manage stress, maintain relationships, and function effectively in daily life (1). Globally, psychiatric conditions—especially anxiety and depression—are among the leading causes of disease burden, with recent studies showing a lifetime prevalence of mental disorders in Saudi Arabia at 34.2% [1,2]. This national survey showed that the prevalence of anxiety and mood disorders was 23.2% and 9.3%, respectively [2]. It also showed that Saudi women had a significantly higher risk of anxiety and mood disorders compared to men [2]. Women, particularly working mothers, are disproportionately affected due to the demands of balancing work, childcare, and family responsibilities [3,4,5].

This mental health crisis is especially pressing in Saudi Arabia, where rapid societal transformation has increased the participation of women in the workforce [5]. Working mothers are at increased risk for psychological distress due to the dual demands of employment and caregiving responsibilities. Mothers in leadership or high-demand roles often face compounded stress due to having limited time for self-care, social support, and mental health maintenance. The dual pressures of professional and domestic life can contribute to elevated levels of anxiety, depression, and emotional exhaustion [3,4,5]. Research has shown that they frequently experience higher levels of stress, emotional exhaustion, and role conflict compared to non-working mothers or women without children [6,7]. These risks may be further exacerbated in cultures where societal expectations of women include being primary caregivers regardless of their employment status. In the Saudi context, although women’s labor force participation is increasing, traditional gender roles remain influential, potentially amplifying the strain experienced by working mothers. This highlights the urgent need for culturally appropriate mental health interventions that support the well-being of this population.

Positive Psychology Interventions (PPIs) offer an innovative, evidence-based strategy to address mental health issues by enhancing positive emotions, engagement, relationships, meaning, and accomplishment—the core components of the PERMA model. These interventions often include practices like gratitude journaling, mindfulness, and strengths-based exercises, which aim not merely to reduce distress but to foster flourishing [8,9,10,11].

Despite their growing popularity in Western settings, PPIs remain underexplored in Arab and collectivist cultures, where social values, religious beliefs, and emotional expression differ significantly from individualistic societies. Gratitude, for example, is a central tenet in Islamic teachings, suggesting potential cultural alignment that could enhance intervention efficacy [12,13,14,15,16]. Nonetheless, it remains unclear whether PPIs developed in Western contexts can be effectively adapted for Saudi mothers without modification.

This study aims to examine the effects of a brief, culturally tailored PPI—consisting of gratitude journaling and well-being workshops—on the mental health of Saudi working mothers in Riyadh. Specifically, it evaluates changes in depression, anxiety, stress, well-being, and gratitude levels before and after the intervention.

## 2. Methods

This study employed a one-group pretest–posttest quasi-experimental design to evaluate the effects of a four-week PPI on the mental health of working mothers. A convenient sample of 37 Saudi working mothers was recruited from a private primary and secondary school in Riyadh, Saudi Arabia. Eligible participants were Saudi nationals, over 18 years of age, employed full-time, and had at least one child.

Participants were invited via an email, which included a brief overview of the study’s purpose. Those interested were provided with detailed information about the study and gave written informed consent prior to participation. The study was approved by the Institutional Review Board of Princess Nourah bint Abdulrahman University (IRB log number: 23-0607).

### 2.1. Intervention Procedure

The intervention lasted four weeks and included two main components: guided gratitude journaling and two structured workshops on positive psychology principles. All intervention materials were provided in Arabic to ensure linguistic and cultural relevance.

1. Gratitude journaling: Participants were asked to write thrice weekly in a guided gratitude journal. Each entry required participants to identify and reflect on three positive experiences or aspects of their day, explain why each was meaningful, and describe how they felt. An example prompt might be: “Write about three things you were grateful for today and why they mattered to you.” Journals were self-administered and submitted electronically each week.
2. Workshops on positive psychology: Two workshops, each lasting approximately two hours, were conducted during the intervention period. The workshops were led by qualified professionals trained in positive psychology and counseling.

**Workshop 1:** Introduced the fundamentals of positive psychology, emphasizing the PERMA model (Positive Emotion, Engagement, Relationships, Meaning, Accomplishment). Participants learned about gratitude’s theoretical basis and practical applications in daily life, including its relevance in Islamic teachings.

**Workshop 2:** Focused on deepening the practice of gratitude. Topics included the benefits of gratitude on mental health, barriers to sustaining a gratitude habit, and culturally grounded practices such as expressing thankfulness in prayer and reflection. Participants engaged in group discussions, role-play, and reflection exercises.

### 2.2. Measures

The survey was self-reported and administered to the mothers from a private school. It consisted of the demographics: age, material status, region, employment, average yearly income, nationality, educational level, number of children, and hobbies. Also, it consisted of three scales that were conducted pre- and post-intervention, including the Depression Anxiety and Stress Scale 21 (DASS-21), the World Health Organization-Five Well-being Index (WHO-5), and the Gratitude Questionnaire (GQ6). In addition, the post-intervention survey also included follow-up questions: frequency of journal writing, perceived benefit of journaling, willingness to continue journaling, and interest in attending future positive psychology courses.

### 2.3. The Depression, Anxiety, and Stress Scale (DASS-21)

We used a validated tool developed by Lovibond and Lovibond (1995) that evaluates depression, anxiety, and stress in adults through 21 items [17]. Participants respond using a 4-point Likert scale ranging from 0 to 3. The total score depression subscale score was subdivided into normal (0–9), mild (10−12), moderate (13−20), severe (21–27), and extremely severe depression (28–42). The anxiety subscale was subdivided into normal (0–6), mild (7–9), moderate (10–14), severe (15–19), and extremely severe anxiety (20–42) [17]. The Arabic version of the scales is valid and reliable [18].

### 2.4. WHO Well-Being Index (WHO-5)

The WHO-5 included five items developed by the World Health Organization to evaluate psychological well-being and was validated in various languages, including Arabic [19,20]. Respondents rate their experiences over the past two weeks on a 6-point Likert scale ranging from 0 to 5. The raw scoring system uses a 0 to represent the worst possible quality of life and a 25 to represent the best quality of life [19,20].

### 2.5. The Gratitude Questionnaire—Six-Item Form (GQ-6)

The GQ-6 is a self-reported, reliable assessment of the propensity to feel grateful, developed by McCullough et al. (2002), and it was validated in Arabic [21,22]. It includes six items, using a 7-point Likert scale ranging from 1 to 7. Items 3 and 6 were reverse-scored.

### 2.6. Statistical Analysis

Data were analyzed using Statistical Package for Social Studies (SPSS 22; IBM Corp., New York, NY, USA). Continuous variables were expressed as the mean ± standard deviation, and categorical variables as percentages. The normality of the pre- and post-intervention scores was assessed using the Shapiro–Wilk test. Wilcoxon’s Signed Rank Test, the Mann–Whitney test, and the Kruskal–Wallis test were used for Continuous variables, and the Chi-square test was used for categorical variables. A *p*-value < 0.05 was considered statistically significant.

## 3. Results

Table 1 shows the demographic characteristics of the participants. The majority of the participants were in the 30 to 40 age group (81.1%), married (89.2%), Saudis (94.6%), and had a bachelor’s degree (70.3%). More than half of the participants work (51.4%) and have an annual family income from SAR 200,000 to 500,000 (55.9%).

**Figure 1:**
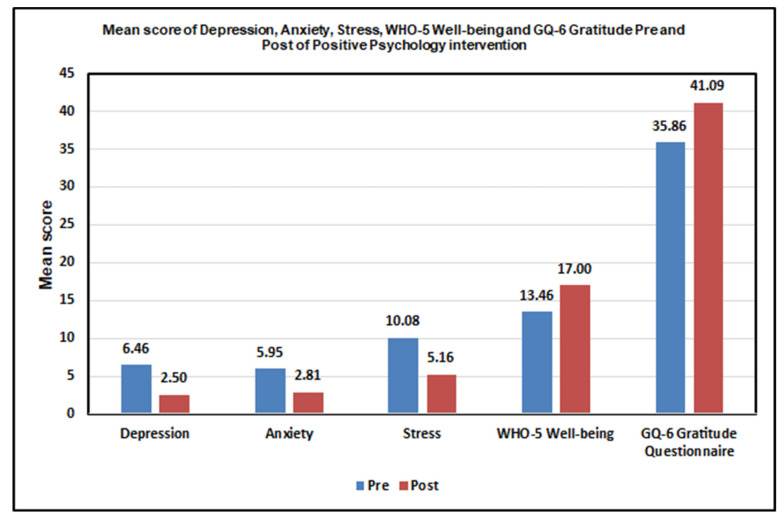
Mean score of depression, anxiety, stress, WHO-5 well-being, and GQ-6 gratitude questionnaire pre- and post-Positive Psychology Intervention.

Table 2 presents the pre- and post-intervention scores for each outcome measure. The results indicate that the PPI significantly decreased depression, anxiety, and stress (*p*-value < 0.001), while enhancing well-being and gratitude significantly (*p*-value = 0.001). Figure 1 illustrates the changes in the levels pre- and post-PPI.

Table 3 shows the prevalence of severity of depression, anxiety, and stress pre- and post-PPI. It is clear that after the PPI, the prevalence of severity of depression and anxiety significantly decreased with (*p*-values < 0.001 and 0.001, respectively).

Table 4 shows the mean scores of depression, anxiety, stress, well-being, and gratitude pre-PPI by the demographic characteristics of the participants. There were no significant differences in the mean scores pre-PPI according to all demographic characteristics.

Table 5 shows the mean scores of depression, anxiety, stress, well-being, and gratitude post-PPI by the demographic characteristics of the participants. There were no significant differences in the mean scores pre-PPI according to all demographic characteristics, except for annual family income, which affected depression and well-being, with *p*-values of 0.022 and 0.038, respectively. Those with annual family income <50,000 SAR have significantly higher levels of depression and lower levels of well-being post PPI.

## 4. Discussion

While PPIs have substantial evidence in Western settings, their applicability in Arab and Muslim-majority contexts is questioned due to cultural, religious, and social factors. Many PPIs are based on individualistic models that may not fit the collectivist, family- and community-oriented values present in Arab cultures. Emotional expression and help-seeking behaviors are shaped by religious beliefs and social norms, possibly limiting acceptance of foreign interventions. Mental health stigma in some segments of society may hinder engagement unless interventions are culturally sensitive. This study investigated the short-term effects of a culturally appropriate PPI on working mothers’ mental health and well-being in Riyadh, Saudi Arabia. The results indicate that the intervention significantly reduced symptoms of depression, anxiety, and stress, while enhancing well-being and gratitude. These findings are consistent with prior literature on PPIs in both Western and emerging non-Western contexts, supporting their potential global relevance [8,9,10,11,16,23,24]

In this study, the positive reception and outcomes suggest that when PPIs are culturally tailored—such as integrating gratitude through both secular and Islamic perspectives—they can resonate well and be effective. This supports evidence that context-sensitive adaptation is crucial for successful global well-being implementation interventions. The intervention’s success can be partially attributed to its cultural and spiritual alignment. Gratitude, a central theme in the workshops and journaling, is deeply rooted in Islamic teachings, where appreciation and reflection are emphasized as virtues. By framing gratitude in both secular and religious terms, the program may have resonated more deeply with participants, enhancing engagement and internalization of the content. This cultural tailoring represents an essential contribution to the emerging literature on context-specific mental health interventions in Arab and Muslim-majority societies [12,13,14,15,25].

While this study showed significant short-term benefits, the long-term effects of PPIs remain an important area for further research. Some studies have found that improvements in well-being can last for several months, especially when participants keep using the skills they learned [23,26]. However, the sustainability of these effects may depend on the length, intensity, and ongoing participation in the intervention. There is no consensus yet on the ideal duration, although interventions usually last 4 to 8 weeks [27]. Future studies should investigate whether periodic “booster” sessions or repeated interventions are necessary to sustain improvements, especially in high-stress groups like working mothers. Long-term studies with follow-up assessments could help determine how durable the effects are and guide best practices for integrating PPIs into routine mental health care.

A notable secondary finding was the differential impact of the intervention based on income levels. Participants from households earning less than SAR 50,000 annually showed significantly higher levels of depression and lower well-being post-intervention compared to higher-income groups. This may suggest that financial stress and reduced access to coping resources could attenuate the benefits of mental health programs. Previous research has shown that individuals with a lower socioeconomic status often face greater structural barriers to mental well-being, including chronic stress, limited social support, and unmet health care needs [28]. Future interventions should consider incorporating tailored modules that address financial stressors or offer additional community-based support for low-income participants.

Importantly, this study contributes to filling a critical gap in the literature by testing PPIs in a Saudi context—an area that has been largely overlooked in global mental health research. While most studies on gratitude-based interventions originate from Western, individualistic cultures, the promising results from this collectivist, Muslim-majority setting suggest that PPIs may be more universally applicable than previously assumed—provided they are adapted thoughtfully.

However, several limitations warrant consideration. The small sample size, lack of a control group, and single geographic location (Riyadh) limit the generalizability of the findings. Additionally, the sample consisted predominantly of well-educated, married women, which may not reflect the broader diversity of Saudi mothers. Furthermore, the intervention duration of only four weeks may have captured only immediate effects; longer-term outcomes remain unknown. Moreover, the study relied solely on self-reported measures, which may be influenced by social desirability or subjective interpretation. While the instruments used (e.g., DASS-21, WHO-5, GQ-6) are validated and widely adopted, they do not replace clinical diagnosis by mental health professionals. The absence of clinician-administered assessments may limit the precision of mental health outcome measurements.

Additionally, the statistical analyses relied on unadjusted comparisons of pre- and post-intervention scores. While the within-subject design controls for some individual variability, it does not account for potential confounding factors such as unmeasured personality traits, concurrent stressors, or differences in adherence to the intervention. These unobserved characteristics may have influenced the outcomes. Future studies should consider using multivariate models or controlled designs (e.g., randomized controlled trials) to better isolate the effects of the intervention and control for potential confounders.

Despite these limitations, this study provides preliminary evidence that brief, culturally tailored PPIs can positively impact the mental health of working mothers in the Saudi context. It also highlights the importance of considering socioeconomic status when designing interventions. Future research should employ randomized controlled trials (RCTs), expand to multiple regions, and examine long-term effects through follow-up assessments.

## 5. Conclusions

This study provides initial evidence that a brief, culturally appropriate Positive Psychology Intervention—combining gratitude journaling and targeted workshops—can significantly improve mental health outcomes among working mothers in Riyadh. The intervention led to meaningful reductions in depression, anxiety, and stress, alongside improvements in well-being and gratitude. These findings demonstrate that Positive Psychology Interventions, when appropriately tailored to local cultural and religious values, hold promise beyond Western contexts.

However, the results also underscore the moderating role of socioeconomic status, suggesting that additional support may be needed for lower-income participants to fully benefit from such interventions. While the findings are encouraging, they must be interpreted within the context of the study’s limitations, including its small, non-random sample, single-group design, and short intervention period.

Future research should build on these insights by conducting randomized controlled trials with more diverse populations across multiple regions and evaluating the long-term sustainability of PPI benefits. Expanding access to low-cost, culturally relevant mental health strategies like these may offer a valuable tool in addressing the growing burden of psychological distress among women in the Arab world.

From a public health perspective, our findings suggest that brief, culturally appropriate PPIs can serve as accessible and preventive strategies to support maternal mental health. Beyond experimental settings, future efforts should explore how these interventions can be embedded into community programs, workplaces, and educational institutions as part of broader behavioral health initiatives. Scaling up through public health campaigns, workplace wellness policies, and school-based mental wellness curricula could facilitate wider access and impact. Such integration into everyday social contexts aligns with national mental health promotion goals and offers a promising avenue for sustainable, population-level interventions. These directions are especially relevant for policymakers and public health planners aiming to reduce the mental health burden among women and families in Saudi Arabia and similar cultural contexts.

## Figures and Tables

**Table 1:** Demographic characteristics of the participants.

		Number	%
**Age**	21–30	1	2.7
	31–40	30	81.1
	41–50	6	16.2
**Social status**	Married	33	89.2
	Divorced	3	8.1
	Single	1	2.7
**Are you currently working?**	Yes	19	51.4
	No	18	48.6
**Annual family income**	<50,000	2	5.9
	200,000–50,000	19	55.9
	>500,000	13	38.2
**Nationality**	Saudi	35	94.6
	Non Saudi	2	5.4
**Educational level**	Secondary	1	2.7
	Bachelor	26	70.3
	Postgraduate	10	27.0
**Number of children**	1–2	17	47.2
	3–4	18	50.0
	4–5	1	2.8
**Do you have a hobby?**	No	7	18.9
	Yes	30	81.1
**Do you express your feelings or events that happened in your day through writing?**	No	27	73.0
	One per week	6	16.2
	2–3 per week	3	8.1
	daily	1	2.7
**How many times have you document the gratitude situation?** (Mean ± SD)		9.29	5.98
**Did you feel that writing down attitudes of gratitude had a positive impact on you during the study period?**	Yes	31	83.8
**Will you continue writing down after the end of school?**	Yes	32	86.5
**Would you like to attend courses in the field of positive psychology in the future?**	Yes	32	86.5

**Table 2:** Mean score of depression, anxiety, stress, WHO-5 well-being and GQ-6 gratitude pre- and post-Positive Psychology Intervention.

Score	Pre		Post		*p*-Value
	Mean	SD	Mean	SD	
Depression (DASS 21)	6.46	4.64	2.50	2.72	<0.001 *
Anxiety (DASS 21)	5.95	5.00	2.81	2.82	<0.001 *
Stress (DASS 21)	10.08	4.73	5.16	3.97	<0.001 *
Well-being (WHO-5)	13.46	4.39	17.00	4.94	0.001 *
Gratitude (GQ-6)	35.86	4.70	41.09	13.45	0.001 *

* Significant *p* value.

**Table 3:** Prevalence of severity of depression, anxiety, stress pre and post positive psychology intervention.

DASS 21	Severity	Pre		Post		*p* Value
		Number	%	Number	%	
Depression	Normal	11	29.7	26	81.3	<0.001 *
	Mild	10	27.0	5	15.6	
	Moderate	11	29.7	0	0	
	Severe	1	2.7	1	3.1	
	Extremely Severe	4	10.8	0	0	
Anxiety	Normal	17	45.9	20	62.5	0.001 *
	Mild	2	5.4	2	6.3	
	Moderate	4	10.8	8	25.0	
	Severe	2	5.4	1	3.1	
	Extremely Severe	12	32.4	1	3.1	
Stress	Normal	12	32.4	24	75.0	0.184
	Mild	5	13.5	3	9.4	
	Moderate	6	16.2	3	9.4	
	Severe	12	32.4	2	6.3	
	Extremely Severe	2	5.4	0	0	

* Significant *p* value.

**Table 4:** Mean score of depression, anxiety, stress, WHO-5 well-being and GQ-6 gratitude pre-Positive Psychology Intervention by demographic characteristics for the participants.

		Depression			Anxiety			Stress			Well-Being			Gratitude		
		Mean	SD	*p* Value	Mean	SD	*p* Value	Mean	SD	*p* Value	Mean	SD	*p* Value	Mean	SD	*p* Value
Age	21–30	3.00	0.00	0.430	2.00	0.00	0.404	10.00	0.00	0.793	9.00	0.00	0.232	35.00	0.00	0.945
	31–40	6.97	4.81		6.40	5.06		10.33	5.03		13.03	4.06		35.97	4.76	
	41–50	4.50	3.51		4.33	4.89		8.83	3.43		16.33	5.32		35.50	5.24	
Social status	Married	6.73	4.65	0.625	6.21	5.09	0.605	10.36	4.89	0.329	13.52	4.60	0.886	35.42	4.76	0.101
	Divorced	5.00	5.20		4.67	4.51		8.00	2.65		13.67	2.52		40.33	1.15	
	Single	2.00	0.00		1.00	0.00		7.00	0.00		11.00	0.00		37.00	0.00	
Are you currently working?	Yes	6.05	3.96	0.939	5.74	4.58	0.915	10.00	4.03	0.855	13.74	4.43	0.772	36.00	4.62	0.951
	No	6.89	5.35		6.17	5.53		10.17	5.49		13.17	4.46		35.72	4.92	
Annual family income	<50,000	6.50	6.36	0.762	7.00	2.83	0.547	9.50	0.71	0.763	12.50	2.12	0.718	40.00	1.41	0.334
	200,000–50,000	5.95	2.90		5.11	4.27		10.37	4.10		13.74	3.63		35.58	5.16	
	>50,0000	7.31	5.68		6.77	6.06		9.77	5.60		11.62	4.50		35.46	4.50	
Nationality	Saudi	6.29	4.59	0.500	6.17	5.04	0.280	10.31	4.76	0.157	13.40	4.47	0.613	36.06	4.75	0.224
	Non-Saudi	9.50	6.36		2.00	1.41		6.00	0.00		14.50	3.54		32.50	2.12	
Educational level	Secondary	9.00	0.00	0.401	3.00	0.00	0.520	13.00	0.00	0.747	10.00	0.00	0.439	29.00	0.00	0.194
	University	7.27	4.87		6.77	5.21		10.42	4.88		13.27	4.36		35.54	5.12	
	Postgraduate	4.10	3.38		4.10	4.23		8.90	4.51		14.30	4.72		37.40	2.76	
Number of children	1–2	5.65	4.33	0.669	4.71	4.24	0.193	8.94	4.62	0.346	13.41	4.76	0.605	36.41	4.70	0.177
	3–4	7.50	5.00		7.61	5.40		11.11	4.84		13.83	4.29		34.94	4.75	
	4–5	6.00	0.00		2.00	0.00		14.00	0.00		10.00	0.00		42.00	0.00	
Do you have a hobby	No	7.57	2.07	0.205	5.57	3.91	0.953	12.00	3.27	0.243	12.29	2.50	0.484	35.86	6.20	0.755
	Yes	6.20	5.05		6.03	5.28		9.63	4.94		13.73	4.72		35.87	4.42	
Do you express your feelings or events that happened in your day through writing?	No	6.52	4.77	0.726	5.96	5.17	0.688	10.30	5.23	0.962	13.48	4.47	0.952	35.81	4.76	0.096
	One per week	7.50	4.51		4.50	4.68		9.50	2.66		13.00	5.44		33.00	3.69	
	2–3 per week	5.33	5.03		7.67	5.69		9.33	5.13		14.00	3.61		41.00	0.00	
	daily	2.00	0.00		9.00	0.00		10.00	0.00		14.00	0.00		39.00	0.00	

**Table 5:** Mean score of DASS 21, WHO-5 well-being and GQ-6 gratitude post-Positive Psychology Intervention by demographic characteristics for the participants.

		Depression			Anxiety			Stress			Well-Being			Gratitude		
		Mean	SD	*p* Value	Mean	SD	*p* Value	Mean	SD	*p* Value	Mean	SD	*p* Value	Mean	SD	*p* Value
Age	21–30	2.00	0.00	0.571	1.00	0.00	0.380	4.00	0.00	0.128	14.00	0.00	0.075	39.00	0.00	0.905
	31–40	2.76	2.93		3.24	2.99		5.84	4.21		16.12	4.64		41.68	15.08	
	41–50	1.50	1.76		1.33	1.51		2.50	1.22		21.17	4.62		39.00	4.65	
Social status	Married	2.18	1.85	0.249	2.71	2.68	0.396	4.79	3.56	0.201	17.57	4.51	0.254	38.61	3.85	0.217
	Divorced	6.33	6.51		4.67	4.16		9.33	6.51		12.67	8.14		64.00	41.57	
	Single	0.00	0.00		0.00	0.00		3.00	0.00		14.00	0.00		42.00	0.00	
Are you currently working?	Yes	2.67	3.25	0.892	3.11	2.78	0.428	5.83	4.46	0.503	16.39	5.97	0.492	42.39	17.85	0.696
	No	2.29	1.94		2.43	2.93		4.29	3.17		17.79	3.21		39.43	3.06	
Annual family income	<50,000	9.50	4.95	0.022 *	7.00	1.41	0.070	12.50	4.95	0.059	8.00	1.41	0.038 *	40.00	0.00	0.892
	200,000–50,000	2.29	1.69		2.82	2.92		5.29	3.46		17.35	4.80		42.71	18.35	
	>500,000	1.60	1.78		2.30	2.54		3.90	3.00		16.50	3.50		39.50	2.92	
Nationality	Saudi	2.50	2.81	0.722	2.87	2.90	0.937	5.23	4.08	0.844	16.73	4.98	0.137	41.17	13.90	1.000
	Non-Saudi	2.50	0.71		2.00	1.41		4.00	1.41		21.00	1.41		40.00	1.41	
Educational level	University	2.87	2.88	0.141	3.04	2.75	0.406	5.43	3.46	0.163	16.74	4.90	0.752	41.35	15.90	0.188
	Postgraduate	1.56	2.13		2.22	3.07		4.44	5.22		17.67	5.27		40.44	2.13	
Number of children	1–2	3.14	3.51	0.752	3.43	2.87	0.609	6.14	5.01	0.583	15.71	6.07	0.612	38.64	3.91	0.576
	3–4	2.19	1.91		2.56	2.85		4.38	2.99		18.25	3.82		43.13	18.71	
	4–5	1.00	0.00		1.00	0.00		6.00	0.00		18.00	0.00		42.00	0.00	
Do you have a hobby	No	5.00	4.74	0.102	5.20	3.42	0.054	6.80	2.77	0.129	14.80	6.30	0.532	40.00	2.92	0.593
	Yes	2.04	1.99		2.37	2.53		4.85	4.12		17.41	4.68		41.30	14.63	
Do you express your feelings or events that happened in your day through writing?	No	2.39	3.01	0.386	2.78	2.95	0.433	5.30	3.75	0.194	16.91	5.09	0.385	41.65	15.76	0.347
	One per week	2.60	0.89		1.60	0.89		3.00	0.71		19.20	3.96		38.20	4.76	
	2–3 per week	2.00	2.65		3.33	2.89		4.00	4.00		16.67	4.16		42.00	0.00	
	daily	6.00	0.00		8.00	0.00		16.00	0.00		9.00	0.00		40.00	0.00	

* Significant *p* value.

## Data Availability

The data presented in this study are available on request from the corresponding author. The data are not publicly available due to privacy and ethical restrictions.
